# Role of mitochondrial dysfunction and dysregulation of Ca^2+^ homeostasis in the pathophysiology of insulin resistance and type 2 diabetes

**DOI:** 10.1186/s12929-017-0375-3

**Published:** 2017-09-07

**Authors:** Chih-Hao Wang, Yau-Huei Wei

**Affiliations:** 1 Center for Mitochondrial Medicine and Free Radical Research, Changhua Christian Hospital, No. 176, 6th Floor, Zhonghua Rd, Changhua City, 500 Taiwan; 2 Institute of Biochemistry and Molecular Biology, National Yang-Ming University, Shih-Pai, Taipei 112 Taiwan; 3 Institute of Biomedical Sciences, Mackay Medical College, Sanzhi, New Taipei City, 252 Taiwan

**Keywords:** Ca2+ homeostasis, Insulin resistance, Metabolic disease, Mitochondrial calcium uniporter, Mitochondria-associated ER membranes, Type 2 diabetes

## Abstract

Metabolic diseases such as obesity, type 2 diabetes (T2D) and insulin resistance have attracted great attention from biomedical researchers and clinicians because of the astonishing increase in its prevalence. Decrease in the capacity of oxidative metabolism and mitochondrial dysfunction are a major contributor to the development of these metabolic disorders. Recent studies indicate that alteration of intracellular Ca^2+^ levels and downstream Ca^2+^-dependent signaling pathways appear to modulate gene transcription and the activities of many enzymes involved in cellular metabolism. Ca^2+^ uptake into mitochondria modulates a number of Ca^2+^-dependent proteins and enzymes participating in fatty acids metabolism, tricarboxylic acid cycle, oxidative phosphorylation and apoptosis in response to physiological and pathophysiological conditions. Mitochondrial calcium uniporter (MCU) complex has been identified as a major channel located on the inner membrane to regulate Ca^2+^ transport into mitochondria. Recent studies of MCU complex have increased our understanding of the modulation of mitochondrial function and retrograde signaling to the nucleus via regulation of the mitochondrial Ca^2+^ level. Mitochondria couple cellular metabolic state by regulating not only their own Ca^2+^ levels, but also influence the entire network of cellular Ca^2+^ signaling. The mitochondria-associated ER membranes (MAMs), which are specialized structures between ER and mitochondria, are responsible for efficient communication between these organelles. Defects in the function or structure of MAMs have been observed in affected tissue cells in metabolic disease or neurodegenerative disorders. We demonstrated that dysregulation of intracellular Ca^2+^ homeostasis due to mitochondrial dysfunction or defects in the function of MAMs are involved in the pathogenesis of insulin insensitivity and T2D. These observations suggest that mitochondrial dysfunction and disturbance of Ca^2+^ homeostasis warrant further studies to assist the development of therapeutics for prevention and medication of insulin resistance and T2D.

## Background

### Regulation of Ca^2+^ homeostasis in metabolism

Ca^2+^ ions are involved in a number of signaling pathways to regulate metabolism, differentiation, proliferation, and life and death of the human cell. Intracellular Ca^2+^ levels should be tightly controlled in response to the timely demands of target cells. This regulation relies on an array of Ca^2+^ channels, transporters and exchangers located on the plasma membrane, the ER and mitochondrial membranes [1].

It has been proven that dysregulation of Ca^2+^ homeostasis is related to metabolic diseases such as obesity, insulin resistance and type 2 diabetes (T2D) in the human and animals. Higher intracellular Ca^2+^ level has been found in primary adipocytes isolated from obese human subjects with insulin resistance [2] and diabetic rats [3]. Besides, increase of serum Ca^2+^ level is positively correlated with the fasting blood glucose and insulin resistance index in the human [4]. Genome-wide association studies (GWASs) revealed that single nucleotide polymorphisms (SNPs) in sarco/ER Ca^2+^ ATPase (SERCA) [5] and inositol 1,4,5-trisphosphate receptors (IP3R) [6], which regulate intracellular Ca^2+^ homeostasis, are associated with the susceptibility to higher body mass index (BMI) and diabetes. Moreover, chelation of Ca^2+^ ions could improve insulin sensitivity of rats fed on the high-fat diet [7].

Many studies have shown that disturbance of Ca^2+^ homeostasis is a key factor in the dysregulation of metabolism. Intracellular Ca^2+^ fluctuation has been substantiated to play a role in the downstream signaling of insulin stimulation. The cytosolic Ca^2+^ level of adipocytes was found to increase upon insulin stimulation [8]. Inhibition of downstream Ca^2+^ signaling either by treatment of calmodulin (CaM) antagonists [8] in adipocytes or by knockdown of IP3R in the primary rat cardiomyocytes [9], respectively, could decrease Glut4 translocation and glucose uptake upon insulin stimulation. Inhibition of Ca^2+^ influx by 2-aminoethoxydiphenyl borate (2-APB), an inhibitor of IP3R and TRP channels, ameliorated insulin-stimulated glucose uptake in skeletal muscle while there was no change in the phosphorylation of Akt [10]. Thus, an increase in the intracellular Ca^2+^ level and the activation of Ca^2+^ sensing proteins may directly or indirectly modulate Glut4 exocytosis, which is the most important step for glucose utilization of muscle cells in response to insulin.

The change in the distribution of some proteins has been demonstrated to play a role in Ca^2+^-mediated insulin action. Recent studies revealed that in adipocytes, synaptotagmin VII (Syt VII) can modulate the translocation of Glut4 and glucose utilization in response to insulin [11]. This finding indicates that Syt VII serves as a downstream sensor of Ca^2+^ signaling to regulate the insulin signaling pathway. Secondly, an actin-binding protein, Myo1c, has been shown to participate in the insulin-stimulated Glut4 translocation, which is regulated by Ca^2+^/CaM signaling because the effect was diminished by treatment with trifluoperazine, a CaM inhibitor [12, 13]. This notion was supported by the finding that phosphorylation of Myo1c by Ca^2+^/CaM kinase II (CaMKII) contributes to insulin-triggered regulation of Glut4 translocation in 3 T3-L1 pre-adipocytes [14]. Moreover, it was demonstrated that FAM3A can facilitate the activation of PI3K/Akt in insulin signaling in liver to improve insulin sensitivity and decrease hepatic gluconeogenesis to control blood glucose in mice [15]. Moreover, activation of Ca^2+^/CaM signaling is required for the FAM3A-mediated Akt activation [15]. In light of the above observations in different cell types and cellular conditions, it is imperative to explore specific Ca^2+^-dependent effectors or Ca^2+^/CaM signaling cascades in the regulation of insulin action under different conditions.

In addition to their role in the action of insulin, Ca^2+^ ions are also involved in adiponectin-mediated regulation of metabolism. Adiponectin has received increasing attention than other adipokines due to the observation that its level is negatively associated with metabolic syndrome and its beneficial effect on cellular bioenergetic metabolism in diabetic mouse models [16, 17]. Briefly, when adiponectin binds to its receptor, AdipoR, in muscle cells, it triggers an increase of Ca^2+^ flux into cytoplasm and activation of Ca^2+^/CaM-dependent protein kinase kinase β (CaMKKβ). In turn, CaMKKβ could further stimulate AMPK activation to induce glucose uptake and β-oxidation of fatty acids. On the other hand, CaMK could also be activated by CaMKKβ, which transcriptionally regulates the expression of PGC-1α to increase the biogenesis and function of mitochondria in muscle cells [17, 18]. These findings suggest that Ca^2+^-dependent signaling cascade is involved in the action of adiponectin to improve not only glucose homeostasis but also lipid metabolism of muscle and other peripheral tissues.

Abundant evidence has substantiated that dysregulation of intracellular Ca^2+^ can cause defects in lipid metabolism in mammalian cells. Functional genetic screens in Drosophila demonstrated the importance of dSERCA and the ryanodine receptor (dRyR) [19], dIP3R [20], and dStim [21] in lipid homeostasis. Recently, abnormal accumulation of lipid droplets was observed in the liver, heart, and skeletal muscle of the SOCE-deficient mice [22]. Fibroblasts isolated from patients with loss-of-function mutations in the *STIM1* or *ORAI1* gene revealed defects in the mobilization of fatty acids from lipid droplets, lipolysis, and β-oxidation of fatty acids [22].

### Mitochondria regulate intracellular Ca^2+^ homeostasis

Mitochondria are able to modulate influx and efflux of Ca^2+^ ions to alter both the amplitude and the spatio-temporal distribution pattern of the intracellular Ca^2+^ levels. The mitochondrial membrane potential produces a large electrochemical gradient (usually between −150 and −200 mV) of the inner membrane of mitochondria so that Ca^2+^ ions can freely cross the outer membrane of mitochondria (OMM). However, there are distinct systems to import or efflux Ca^2+^ through the inner membrane of mitochondria (IMM). Mitochondrial Ca^2+^ uniporter machinery facilitates the entry of Ca^2+^ ions to the matrix. H^+^/Ca^2+^ and Na^+^/Ca^2+^ exchangers (NCX) efflux Ca^2+^ ions from matrix to the cytosol. Tight regulation of these proteins is important to increase the Ca^2+^ level to activate mitochondrial enzymes and to prevent accumulation of Ca^2+^ ions and Ca^2+^ overload within the mitochondria [23].

The influx and efflux rates of Ca^2+^ between mitochondria must be balanced. Disruption of this balance may result in the opening of the mitochondrial permeability transition pore (mPTP) and the induction of cell death [24]. Ca^2+^ ions taken up into the mitochondrial matrix can increase ATP production via Ca^2+^-dependent activation of three important metabolic enzymes in the matrix, which include the pyruvate dehydrogenase (PDH), α-ketoglutarate dehydrogenase (αKGDH) and isocitrate dehydrogenase (IDH) [25]. The mitochondrial Ca^2+^ uptake will affect Ca^2+^ signaling at local and the global levels. The Ca^2+^ ions released through the activation of IP3 receptor of ER in response to external stimuli can activate a series of signal transductions, but these activations need to be shut down at the right moment by sequestration of Ca^2+^ ions into mitochondria. This regulation highly depends on the efficiency of the functional coupling between mitochondria and ER and on the subcellular distribution of mitochondria [26]. Thus, the buffering capacity of Ca^2+^ ions by mitochondria plays a crucial role in the modulation of the Ca^2+^-dependent signaling and in the pathophysiology of a wide spectrum of diseases [27, 28].

### Mitochondrial calcium uniporter complex in human cells

**Fig. 1:**
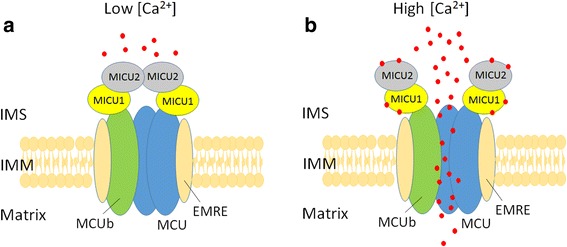
Mitochondrial calcium uniporter complex and the regulation of the entry of Ca^2+^ ions into mitochondria. The protein complex of mitochondrial calcium uniporter is composed of the pore-forming proteins (MCU, MCUb, EMRE), and the regulatory proteins (MICU1, MICU2). The regulation of the entry of Ca^2+^ ions by mitochondrial calcium uniporter complex is demonstrated here. **a** When the concentration of Ca^2+^ ions is low in the IMS, the heterodimer of MICU1 and MICU2 blocks the channel of MCU to inhibit the entry of Ca^2+^ ions. **b** When the Ca^2+^ ions level is high upon stimulation, binding of Ca^2+^ ions to the MICU protein elicits a conformational change to open the channel, resulting in the transport of Ca^2+^ ions into mitochondria to activate several dehydrogenases in the matrix of mitochondria. IMS, intermembrane space; IMM, inner mitochondrial membrane

Mitochondrial calcium uniporter complex, a highly selective channel responsible for Ca^2+^ uptake of mitochondria, consists of both pore-forming and regulatory subunits (Fig. 1). Human mitochondrial calcium uniporter (MCU) complex has been identified as a large protein complex (~480 kDa) in the intensive studies of past few years [29, 30]. MCU is composed of two coiled-coil domains and two transmembrane domains and is the main channel for Ca^2+^ uptake [31, 32]. The other two pore-forming proteins are MCUb [33] and essential MCU regulator (EMRE) [34]. It has been shown that MCU per se is sufficient to execute the Ca^2+^ uptake. MCUb shares a 50% similarity with the MCU but the difference of some amino acids in the pore forming region makes it an inhibitory subunit [33]. EMRE was just identified by SILAC-based quantitative mass spectrometry in 2013 by Sancak et al. [34]. Recently, EMRE has been demonstrated as a matrix Ca^2+^ sensor and its interaction with MICU1 contributes to collaborative regulation of the Ca^2+^ uptake current of the MCU complex. Deletion of its matrix-localized acidic C-terminal domain abolished the regulation, causing an increase of Ca^2+^ uptake from MCU [35].

Mitochondrial calcium uptake proteins (MICU) in the intermembrane space of mitochondria have been identified as regulatory proteins to control the Ca^2+^ ion transport through the MCU. There are three MICU proteins in human cells and all of them contain the EF hand domain for Ca^2+^ binding [36]. MICU1 and MICU2 are ubiquitously expressed in mammalian tissues, but MICU3 is restricted to the central nervous system [37]. MICU2 forms an obligate heterodimer with MICU1 through a disulfide bond that interacts with MCU. A model proposed for the regulation of Ca^2+^ uptake in human cells is described in Fig. 1. Briefly, when Ca^2+^ ion concentration in the intermembrane space of mitochondria is low, the heterodimer of MICU1 and MICU2 blocks the MCU channel to prevent uptake of Ca^2+^ ions by mitochondria. When there is an increased release of Ca^2+^ ions from ER or import from extracellular compartments, the elevation of cytosolic level of Ca^2+^ ions will increase the binding of Ca^2+^ ions to MICU proteins. Upon increase of cytosolic Ca^2+^ ions, the inhibition of MCU is relieved due to the conformational change of MICU1 and MICU2 after Ca^2+^ ion binding, and Ca^2+^ ions could then be transported through the MCU [38].

### Dysregulation of mitochondrial Ca^2+^ ions in human diseases

One of the physiological roles of the MCU complex has been established in the control of ATP production through activation of Ca ^2+^-dependent dehydrogenases in the mitochondrial matrix, modulation of the duration of cytosolic Ca^2+^ signals by buffering cytosolic Ca^2+^ ions. The identification of the molecular components of the uniporter provides an unprecedented opportunity to unravel the role of mitochondrial Ca^2+^ ions in the regulation of cellular metabolism in more detail using genetic tools (Table 1).

**Table 1:** The role of mitochondrial Ca^2+^ homeostasis in cellular functions

Study subjects	Manipulation of mitochondrial Ca^2+^ ions	Observations	Ref.
In vitro			
Human			
HeLa cells	knockdown of MCU	increase of mitochondrial Ca^2+^	[40]
		increase of ROS	[40]
		decrease of SOCE response	[43]
Lung cells	knockdown of MCU	decrease of inflammasome activation	[44]
		decrease of ROS	[44]
Skin fibroblasts	point mutation of MICU1	decrease of maximal OCR	[47]
		increase of mitochondrial Ca^2+^ uptake	[47]
HEK cells	C-terminal deletion of EMRE	increase of mitochondrial Ca^2+^	[35]
Hepatocytes	knockdown of MAMs components (IP3R, VDAC, GRP75)	decrease of insulin signaling	[69]
Rat			
Beta cells	knockdown of MCU or MICU1	decrease of mitochondrial Ca^2+^	[41]
		decrease of glucose-stimulated insulin secretion	[41]
Leukemia cells	knockdown of MCU	decrease of SOCE response	[42]
		decrease of mitochondrial Ca^2+^ uptake	[42]
Cardiomyocytes	overexpression of TFAM	increase of mitochondrial Ca^2+^	[61]
		increase of ATP production	[61]
		increase of SERCA expression	[61]
Mouse			
Adipocytes	downregulation of TFAM, PGC-1α	decrease of mitochondrial Ca^2+^	[62]
		increase of ROS	[62]
		decrease of insulin-stimulated glucose uptake	[62]
In vivo			
Mouse			
Skeletal muscle	knockout of MCU	decrease of mitochondrial Ca^2+^ uptake	[39]
		decrease of maximal OCR	[39]
		decrease of PDH activity	[39, 46]
		decrease of muscle function	[39]
		decrease of muscle size	[46]
		defects in mitochondrial morphology	[46]
Heart	overexpression of DN-MCU	decrease of maximal OCR	[45]
		decrease of heart rate upon stimulation	[45]
Adipose tissue	knockdown of MAMs components (Cisd2)	glucose intolerance	[60]
		decrease of maximal OCR	[60]
		decrease of mitochondrial Ca^2+^ uptake	[60]
Liver	knockout of MICU1	increase of mitochondrial Ca^2+^	[48]
		increase of ROS	[48]
		decrease of ATP	[48]
		defects in mitochondrial morphology	[48]
	knockdown of MICU1	impaired liver regeneration	[49]
	inflexibility of MAM structure	decrease of maximal OCR	[72]
		decrease of glucose infusion rate	[72]
		glucose intolerance	[72]
	knockdown of MAMs components (CypD)	hepatic insulin resistance	[69]

It is accepted that MCU plays a role in excitation-energetic coupling through the activation of mitochondrial matrix dehydrogenases. Manipulation of components in the MCU complex could alter the activity of the PDH complex and intracellular ATP levels of human cells. Ca^2+^-sensitive PDH phosphatase (PDP) activated by Ca^2+^ ions in the matrix can dephosphorylate PDH and increase its activity. The MCU complex activity is positively correlated with the PDH activity and oxidative phosphorylation in mitochondria. A deficiency of MCU in skeletal muscle resulted in an increase of phosphorylation of PDH and concomitant decrease of PDH activity [39]. Knockdown of MICU1, which led to an increase of basal levels of Ca^2+^ ions in the mitochondrial matrix, decreased the phosphorylation and increased the activity of PDH in HeLa cells [40]. Furthermore, ablation of MCU in pancreatic β cells exhibited a decrease of intracellular ATP concentration following glucose stimulation [41]. This resulted in diminished glucose-stimulated insulin secretion [41]. Thus, the above-mentioned in vitro studies have provided compelling evidence to substantiate that MCU plays a role in excitation–energetic coupling.

It has been shown that alteration of the MCU complex is involved in regulating transient fluxes of cytosolic Ca^2+^ ions to modulate the cellular metabolism. It was demonstrated that Ca^2+^ signaling downstream of the leukotriene receptor is influenced by MCU [42]. In rat basophils, knockdown of MCU resulted not only in defective mitochondrial Ca^2+^ uptake but also in the suppression of Ca^2+^-dependent gene expression following stimulation of the leukotriene receptor with leukotriene C4 (LTC4). The MCU seems to involve in two processes that are relevant to the immune signaling: store-operated calcium entry (SOCE) and activation of the NLR family pyrin domain containing 3 (NLRP3) inflammasome. Lack of MCU has been shown to reduce the SOCE response after inositol trisphosphate-mediated Ca^2+^ ions release from ER [43], which is the underlying cause of defects in the activation of the NLRP3 inflammasome induced by *Pseudomonas aeruginosa* in airway epithelial cells from patients with cystic fibrosis [44]. Taken together, these different lines of evidence confirm a role for the MCU uniporter in cellular Ca^2+^ signaling and substantiate its importance in the immune response.

MCU deficiency can be tolerated in mice with a mixed genetic background. MCU knockout was found to be lethal in C57BL/6 mice, whereas the knockout mice with an outbred CD1 background were viable. MCU-knockout CD1 mice displayed no obvious phenotype but exhibited impaired tolerance to exercise. This is consistent with a cellular role of MCU in stimulating the activity of the TCA cycle during Ca^2+^ signaling events associated with muscle contraction [39]. In addition to whole-body MCU knockout, the overexpression of a dominant-negative MCU protein in sinoatrial node cells in mice also revealed a link between the Ca^2+^ uniporter activity and cellular energetics [45]. Although there was no significant difference at base line between wild-type and mutant animals, the heart rate was unable to increase in mutant mice in response to β adrenergic agonists. This observation revealed an important role of the MCU complex in the ‘fight-or-flight’ response of the animals. Skeletal muscle cells infected with adeno-associated viral vectors (AAVs) was used to create the overexpression or knockdown of MCU in the tissue specific manner. Overexpression of MCU triggered skeletal muscle hypertrophy during post-natal development and knockdown of MCU led to muscle atrophy in adulthood [46]. Notably, MCU overexpression could protect muscle tissues from the loss of muscle mass upon denervation, indicating a potential therapeutic role of MCU modulation in muscle atrophy [46]. Taken together, these findings demonstrate the physiological importance of MCU as the major mammalian Ca^2+^ uniporter, including its role in skeletal muscle contraction and in the response of cardiac muscle to adrenergic stimulation.

Loss of MICU1 expression by truncating mutations in the human could lead to skeletal muscle myopathy, learning disability and movement disorder [47]. The pathological phenotypes caused by the loss of MICU1 manifests in a tissue-specific manner, which is reminiscent of mitochondrial disorders. Deficiency of the MICU1 could result in an increase of perinatal mortality in mice [48, 49]. The surviving mice showed ataxia and muscle weakness which is similar to afflicted patients. MICU1 KO led to Ca^2+^ overload in mitochondria and increase of ROS production. Interestingly, the impairment of Ca^2+^ regulation could be restored by age-dependent decline of the EMRE expression. This indicates that the remodeling of MCU complex may help to maintain the Ca^2+^ homeostasis [48]. Hepatocyte-specific MICU1 knockdown by the injection of AAV-Cre did not reveal significant gross defect in liver but the liver was unable to regenerate from injury. In addition, MICU1-deficienct hepatocytes were found to be more susceptible to the opening of mitochondrial permeability transition pores (mPTP) [49]. Future studies are warranted to answer the questions as to whether patients with mutations in MICU1 have pathological involvement of other organ systems, especially in tissues with high energy-demand or high mitochondrial content like brain, liver, and brown adipose tissue.

### Mitochondria-nuclear crosstalk via Ca^2+^ signaling

The retrograde signals from mitochondria can trigger gene transcription in the nucleus to induce adaptive responses or modulate cellular metabolism [50]. Although the reactive oxygen species (ROS) production in mitochondrial respiration has been known as putative retrograde signaling molecules linking mitochondrial dysfunction to insulin insensitivity [51], the emerging evidence has substantiated the importance of other known mitochondrial retrograde signals. Recent studies have pointed out the crucial role of Ca^2+^ signaling from mitochondria in the regulation of cell metabolism. Dysregulation of intracellular Ca^2+^ homeostasis due to ATP depletion and release of Ca^2+^ ions from the mitochondria have been proposed as a principal cause for insulin resistance, but no detailed studies have been performed yet on the mitochondrial and cellular Ca^2+^ transport processes to clarify this issue. Although it is established that transcriptional control of metabolism by Ca^2+^ is exerted indirectly via Ca^2+^-dependent kinases and phosphatases, such as CaMK and calcineurin, which regulate the expression of PGC-1α [52], the underlying mechanism that generates the retrograde signals remains to be determined. It is important to answer the questions as to whether the feedback regulation between mitochondria and the nucleus is effected through the cellular and mitochondrial Ca^2+^ signaling networks and what are the components involved in these processes.

Many studies have revealed that an increase in the intracellular Ca^2+^ level can inhibit the differentiation and maturation of human mesenchymal stem cells and 3 T3-L1 preadipocytes. For example, increases of the intracellular Ca^2+^ level by incubation of adipocytes in the culture media containing a high concentration of Ca^2+^ ions [53], activation of Ca^2+^ ion channels or receptors [54, 55], and inhibition of SERCA by thapsigargin [56] have been demonstrated to interrupt the adipogenic differentiation signaling in 3 T3-L1 preadipocytes. Ca^2+^-dependent enzymes including calcineurin (CaN), a Ca^2+^-dependent phosphatase, CaMKII, a Ca^2+^/CaM kinase 2 as well as calreticulin (Calr), a Ca^2+^-buffering chaperone in the ER, have been demonstrated, respectively, to play important roles in adipogenesis [57–59]. In a very recent study, we showed that mitochondrial dysfunction induced by Cisd2 deficiency increased the cytosolic level of Ca^2+^ ions and activated Ca^2+^-calcineurin-dependent signaling, which inhibited the transcriptional cascades at the late stage of adipogenesis in mice [60]. These findings indicate that the maintenance of Ca^2+^ homeostasis and normal mitochondrial function by Cisd2 are essential for adipogenic differentiation and function of adipocytes, which in turn regulates systemic glucose homeostasis in mice. Dysregulation of Ca^2+^ homeostasis and insulin insensitivity could be similarly induced in mouse progenitor cells-derived adipocytes with genetic manipulation of TFAM [61] or down-regulation of PGC-1α expression [62]. These genetic approaches have provided different lines of evidence to support the notion that disturbance of Ca^2+^ homeostasis caused by mitochondrial dysfunction plays an important role in T2D and insulin resistance in mice. Regulation of mitochondrial Ca^2+^ ions also modulates the morphology of the skeletal muscle. MCU overexpression by adeno-associated viral vectors induced muscle hypertrophy and MCU silencing triggered muscle hypotrophy in mice [46]. The control of muscle size involves the regulation of the expression of a set of genes by IGF-AKT and PGC-1α signaling cascades [46]. In addition, RNA microarray analyses demonstrated that modulation of the activity of MCU could control the global gene expression, thereby led to the identification of a Ca^2+^-dependent mitochondria-to-nucleus route that links mitochondrial function to the control of muscle mass [63].

### Mitochondria-associated ER membranes (MAMs)

Mitochondria-associated ER membranes (MAMs) are the contact sites between the mitochondrial outer membrane and ER membrane, which are defined as structural membranes between the two organelles [26]. This special intracellular membrane structure is crucial for an accurate and efficient communication and transport of Ca^2+^ ions between the two organelles, which are the two largest Ca^2+^ storage sites in human cells. MAMs are responsible for dynamic and efficient transmission of physiological and pathological Ca^2+^ signals between the ER and the mitochondria. Due to the enrichment of Ca^2+^ handling proteins present in the MAMs, the functional coupling at the ER-mitochondria interface is very important for the regulation of intracellular Ca^2+^ homeostasis during metabolic reprogramming and cellular adaptation to various physiological and environmental stimuli [64]. In addition, it has been suggested that MAMs serve as an integrator of energy metabolism because of the enrichment in MAMs of functionally diverse enzymes involved in the metabolism of glucose and fatty acids [65, 66].

### Alterations of ER-mitochondria coupling contributes to insulin resistance in obesity and diabetes

Defects in MAMs have been suggested to play a role in the pathogenesis of diseases such as Alzheimer’s disease, insulin resistance and T2D [64, 67, 68]. An in situ proximity ligation assay (PLA) was developed to visualize and quantify the ER-mitochondria connections by monitoring the interactions between VDAC1-IP3R1, Grp75-IP3R1 and CypD-IP3R1, respectively. Using this technique, the disruption of MAMs integrity could be observed in primary hepatocytes from the obese mice or palmitate-induced insulin resistance in the mouse or cultured cells [69]. Knockdown of IP3R1 to reduce MAMs structure could trigger mitochondrial dysfunction and glucose intolerance in obese mice. In addition, it was found that diabetic mice treated with rosiglitazone or metaformin not only reinforce MAMs integrity but also improve insulin sensitivity and glucose homeostasis [69]. Restoration of MAMs by overexpression of Grp75 could also improve insulin sensitivity in palmitate-treated primary culture of hepatocytes [69]. Primary cultures of hepatocytes and HuH7 cell line recapitulated the phenotype of insulin resistance in media containing high concentrations of glucose, which was associated with the decrease of interactions in MAMs, mitochondrial fragmentation, decrease of the dynamics and respiration rate of mitochondria [70]. Mechanistically, hepatocytes cultured in media containing high concentration of glucose exhibited an increase of flux through pentose phosphate pathway and activation of protein phosphatase 2A (PP2A) [70]. On the other hand, high glucose also decreased the transport of Ca^2+^ ions to mitochondria and increase of cytosolic level of Ca^2+^ ions, which could further activate PP2A. Thus, inhibition of PP2A by okadaic acid could prevent high glucose-induced disruption of MAMs and restored the morphology and bioenergetic function of mitochondria [70]. Impairment of ER-mitochondria interactions and abnormality of Ca^2+^ homeostasis have also been observed in the liver of mice with deficiency of cyclophilin D (CypD), which is a mitochondrial protein that regulates mPTP and was recently found in MAMs fractions. Conversely, restoration of MAMs integrity by overexpression of CypD could improve insulin sensitivity and insulin signaling cascade [71]. In contrast, abnormal chronic increases in the formation of MAMs resulted in mitochondrial Ca^2+^ ions overloading, which could impair the mitochondrial bioenergetic function and increase the ROS production in the liver of obese mice [72]. Although the discrepancy still exists as to whether increase or decrease of MAMs structure is better for the regulation of Ca^2+^ homeostasis, the common conclusion is that MAMs structure should be flexible and dynamic for an efficient control of the Ca^2+^ level in response to stimuli or the change of nutrients.

It has been reported that Cisd2 is localized on both ER and mitochondrial membranes [73, 74]. Cisd2 deficiency could lead to an alteration of Ca^2+^ ions level in the ER [74]. Recently, we provided evidence to show that direct interactions exist between Cisd2 and Gimap5 on the MAMs and thereby modulate the mitochondrial uptake of Ca^2+^ ions, which in turn regulate the intracellular Ca^2+^ homeostasis. This novel role of Cisd2 in MAMs is crucial for adipogenic differentiation and function of adipocytes, and even in the glucose tolerance and insulin sensitivity of the mouse [60]. Taken together, these observations suggest the importance of MAMs in the regulation of Ca^2+^ level and mitochondrial function, which may participate in the modulation of glucose homeostasis and insulin sensitivity. It is worth mentioning that MAMs formation is a dynamic process to support efficient transmission of Ca^2+^ ions and lipid biosynthesis, which culminates in an increase of mitochondrial function to meet the cellular energy demand under stress conditions. The fluctuating feature of MAMs in cooperation between ER and mitochondria provides an inter-organelle communication for tissue cells to adapt to specific physiological and environmental conditions.

## Conclusion

**Fig. 2:**
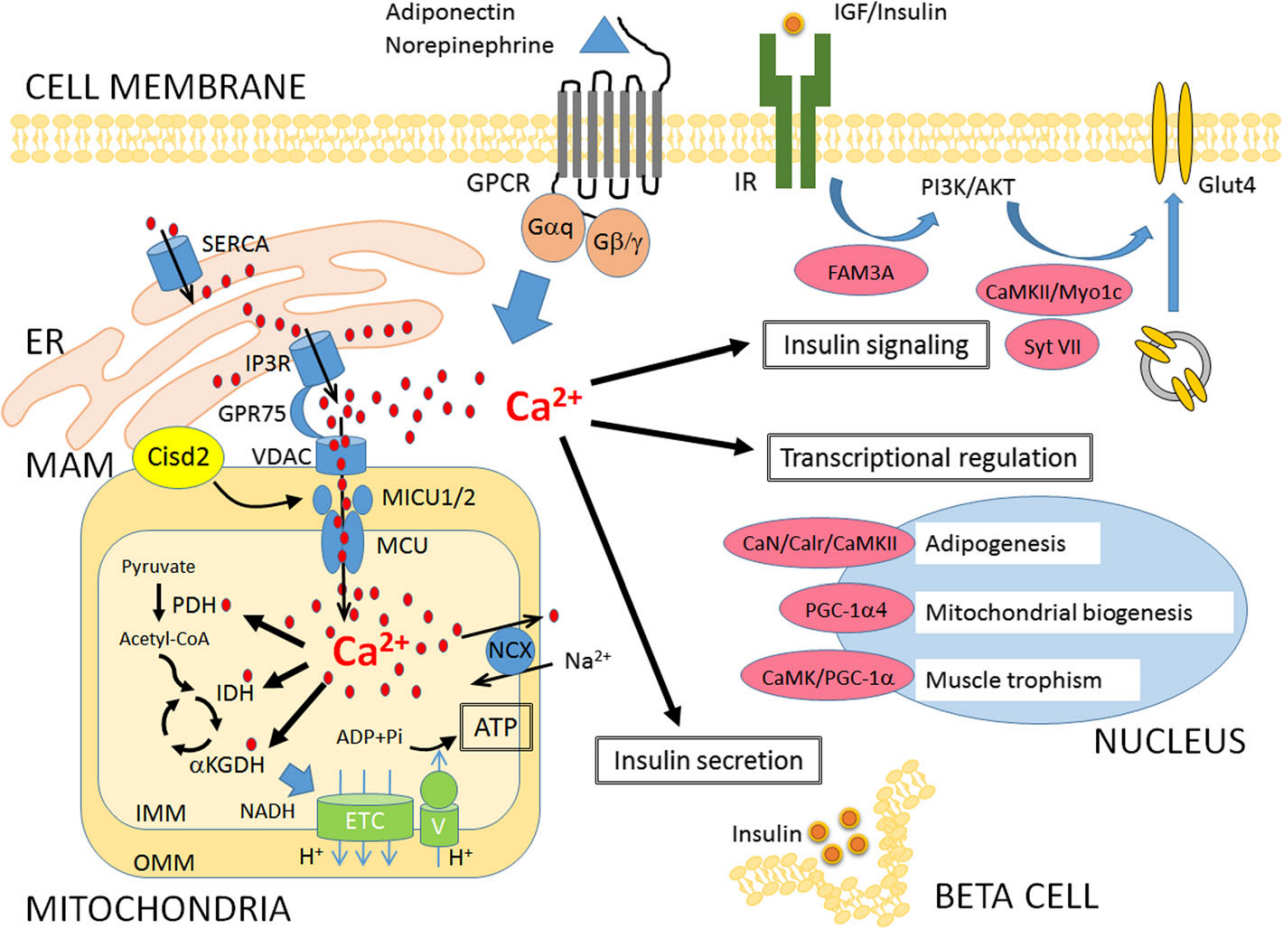
Illustration of the role of defects in mitochondria-mediated regulation of Ca^2+^ homeostasis in the pathogenesis of insulin resistance and type 2 diabetes. The intracellular level of Ca^2+^ ions in a normal human cell is regulated and maintained within a small range of concentration. The fluctuation of the level of Ca^2+^ ions from extracellular influx or release of intra-organelle leads to activation of Ca^2+^-dependent signaling to alter the gene expression or protein trafficking in response to the stimulation (i.e., adiponectin or norepinephrine). Increase of cytosolic level of Ca^2+^ ions initiates the activation of insulin signaling and transcriptional regulation in insulin-responsive tissues such as adipocytes and muscle. On the other hand, Ca^2+^ ions can facilitate insulin secretion in beta cells. All of these effects are beneficial to glucose utilization and insulin sensitivity in the human body. For instance, the Ca^2+^-dependent activation of FAM3A improves phosphorylation of AKT and the activation of CaMKII or synaptotagmin VII (Syt VII) allow efficient translocation/docking/fusion of glucose transporter 4 (Glut4) to the plasma membrane in insulin- responsive cells upon insulin stimulation. Moreover, Ca^2+^ homeostasis also regulates gene transcription to affect adipogenesis, muscle trophism, and mitochondrial biogenesis through Ca^2+^-dependent activation of a number of proteins. Mitochondria modulate intracellular Ca^2+^ homeostasis by its high capacity of Ca^2+^ uptake through the MCU complex and interaction with ER via the MAMs structure. Mitochondrial Ca^2+^ uptake plays as a role in the buffering of cytosolic Ca^2+^ ions and in the boost of the ATP production. Three enzymes (PDH, IDH, αKGDH) involved in oxidative metabolism are regulated by Ca^2+^ ions directly or indirectly, providing more NADH to the electron transport chain (ETC). Mitochondrial dysfunction disrupts intracellular Ca^2+^ homeostasis and leads to dysregulation of the above-mentioned Ca^2+^-dependent signaling events and impairment of glucose utilization and insulin response in the affected cells. Ultimately, these abnormalities will culminate in insulin insensitivity of target tissue cells and thereby develop T2D

This review has provided an overview of recent advances in the role of mitochondrial dysfunction and dysregulation of intracellular Ca^2+^ homeostasis in the pathogenesis of metabolic diseases such as insulin resistance and T2D (Fig. 2). We have especially focused on the dysregulation of intracellular Ca^2+^ homeostasis caused by functional defects in the MCU complex, which is located on the inner membrane of mitochondria. Although overproduction of ROS and defects in lipid metabolism have been established as a common cause of T2D and insulin resistance, the defects in the maintenance of intracellular Ca^2+^ levels by mitochondria deserves proper attention. In addition, mitochondrial Ca^2+^ has been well documented in the contribution of ROS production within mitochondria [75]. Given that mitochondria are intracellular organelles involved in the execution of many cellular functions and that there are multiple pathways involved in the regulation of metabolism, in-depth studies of the effects of mitochondrial dysfunction on Ca^2+^ homeostasis are warranted to gain a better understanding of the complex pathophysiology of metabolic disorders.

After identification of the MCU complex, the key regulator of the mitochondrial Ca^2+^ signaling, a new area of research has emerged. Molecular genetic manipulation and development of transgenic animal models have allowed us to directly address exciting issues of mitochondrial Ca^2+^ signaling in the pathophysiology of diseases associated with mitochondrial dysfunction. In the past decade, we have witnessed the advances in a better understanding of the roles of Ca^2+^ transporters in the regulation of Ca^2+^ homeostasis, mitochondrial bioenergetics and even in metabolic reprogramming. However, many aspects of mitochondrial dysfunction in the pathogenesis of diseases await further investigation. Until now, the stoichiometry and oligomeric state of each of the components of the MCU complex, the major mitochondrial Ca^2+^ uniporter, and the dynamic change of their stoichiometry have remained unknown. Elucidation of the composition of the MCU complex in different cell types at distinct developmental stages is most important. The expression levels of specific component in the MCU complex have been determined in different tissues and cell lines. Some studies have shown that the relative expression levels of MCU and its interaction partner proteins are in line with the predicted mitochondrial Ca^2+^ uptake behavior. However, we do not exclude the possibilities that other regulatory systems may contribute to the regulation of the MCU activity. In addition, what kinds of signaling or stimuli that contribute to transcriptional regulation of genes in the MCU complex are still unclear. Interestingly, the alteration in the expression ratio between MCU and its negative-dominant MCUb in different types of tissues suggests that it might contribute to the spatiotemporal control of mitochondrial uptake of Ca^2+^ ions and Ca^2+^-dependent activation of mitochondrial function. Given that protein modification can rapidly regulate the function, interaction, and conformational change of proteins, work has to be done in the future on the post-transcriptional regulation of the function of the MCU complex, which certainly plays an important role in the cellular response to external stimuli and physiological signals.

Most importantly, we discuss in this review the importance of mitochondria-ER cross-talk in the maintenance of Ca^2+^ homeostasis and suggest that dysregulation of this inter-organelle communication may play a key role in the pathogenesis of insulin insensitivity and T2D. Lack of Cisd2, an iron-sulfur protein localized in the MAMs, significantly affects this inter-organelle communication and alters the Ca^2+^ buffering capacity of mitochondria in adipocytes. Moreover, recent studies demonstrated that ER-mitochondria interactions were decreased in diabetic mice and in primary culture of hepatocytes and in HuH7 cells that had been cultured in a high-glucose medium or treated with palmitate. These findings indicate that the structural integrity of MAMs may contribute to the maintenance of Ca^2+^ homeostasis. It is thus important to determine the dynamic properties of MAMs in different type of cells under different cellular context and physiological conditions. When addressing the communication between the two organelles, the reciprocal effects on Ca^2+^ homeostasis from each other should be considered. Further studies are warranted to elucidate the cross-talk and responses between defective mitochondria and ER. It is imperative to clarify whether there are concomitant beneficial effects for ER when adipocytes are treated with mitochondria-targeting antioxidants (such as mito-CoQ_10_). The insight gained from studies of the inter-organelle communications can help us better understand the pathogenesis of the complicated and multifactorial disorders such as T2D. This line of research will also provide us novel information for the development of therapeutic agents to improve the function and/or structural integrity of MAMs. We have demonstrated that dysregulation of Ca^2+^ homeostasis is a novel mechanism underlying the mitochondrial dysfunction-related insulin insensitivity of adipocytes and possibly an etiology factor of T2D. We believe that simultaneous improvement of the structure and function of mitochondria and ER may be a useful strategy to restore and maintain glucose homeostasis in the human and animals.

## Abbreviations

2-APB: Aminoethoxydiphenyl borate
AAVs: Adeno-associated viral vectors
AdipoR: Adiponectin receptor
BMI: Body mass index
Calr: Calreticulin
CaM: Calmodulin
CaMKII: Ca^2+^/CaM-dependent protein kinase II
CaMKKβ: Ca^2+^/CaM-dependent protein kinase kinase β
CaN: Calcineurin
EMRE: Essential MCU regulator
GWASs: Genome-wide association studies
IDH: Isocitrate dehydrogenase
IMM: Inner mitochondrial membrane
IP3R: Inositol 1,4,5-trisphosphate receptors
LTC4: Leukotriene C4
MAMs: Mitochondria-associated ER membranes
MCU: Mitochondrial calcium uniporter
MICU: Mitochondrial calcium uptake proteins
mPTP: Mitochondrial permeability transition pore
NCX: Na^+^/Ca^2+^ exchangers
NLRP3: NLR family pyrin domain containing 3
OMM: Outer mitochondrial membrane
PDH: Pyruvate dehydrogenase
PDP: PDH phosphatase
ROS: Reactive oxygen species
RyR: Ryanodine receptor
SERCA: Sarco/ER Ca^2+^ ATPase
SNPs: Polymorphisms
SOCE: Store-operated calcium entry
Syt VII: Synaptotagmin VII
T2D: Type 2 diabetes
TCA cycle: Tricarboxylic acid cycle
αKGDH: α-ketoglutarate dehydrogenase
